# Supplementary material for: Content-rich biological network constructed by mining PubMed abstracts
Source: BMC Bioinformatics. 2004 Oct 8;5:147. doi: 10.1186/1471-2105-5-147 (PMC528731; doi:10.1186/1471-2105-5-147)
Supplement: Additional File 5 — The original Chilibot query results of the term "long-term potentiation (LTP)" and 22 other terms, limiting the latest references analyzed to the years 1990, 1995, 2000, and 2004. [file 1471-2105-5-147-S5.bz2 › chilibotAdditionalFile5/ltp2000/html/ATF_CAMKIV.html]

 


 **ATF** and **CAMKIV** 
  
Found 2 abstracts in PubMed,  **2 abstracts were retrieved and analyzed**.  


---

 Search Google  |
 PDF files only 
|  EDU domain only 

---

**Interactive relationship** (e.g. stimulation, inhibition, etc)

- The  **CaMKIV**  mediated gene induction requires the activity of CREB  **ATF**  family members but is independent of PKA activity.  Ref: 8065343 Mol Cell Biol, 1994
- Mutagenesis studies showed that Thr69, Thr71, and Thr73 of  **ATF**  2 are all required for activation by  **CaMKIV** .  Ref: 10909971 Diabetes, 2000

- :-)

  **Parallel relationship** (e.g. studied together, co-existance, homology, etc.)

  - **CaMKIV**  induced  **ATF**  2 transcriptional activity was not altered by activation of cJun NH2 terminal protein kinase or p38 mitogen activated protein kinase.  Ref: 10909971 Diabetes, 2000
